# Prevalence of genitourinary infection in diabetic patients treated with SGLT 2 inhibitors

**DOI:** 10.4314/ahs.v23i1.29

**Published:** 2023-03

**Authors:** Acharya Shrikrishna, Bhat Archana

**Affiliations:** 1 KS Hegde Medical Academy, Endocrinology; 2 Father Muller Medical College, department of medicine

**Keywords:** Diabetes Complications, SGLT 2 inhibitors, genitourinary infections

## Abstract

**Introduction:**

Genitourinary infections are common in Diabetes patients compared to the general population more so in patients with Sodium glucose co transporter 2 inhibitors (SGLT2i) treatment , So, we did a study to find the prevalence of genitourinary infection in T2DM patients treated with SGLT2i.

**Methods:**

One hundred and twenty patients receiving SGLT2i, who had signs and symptoms indicative of genitourinary infections were enrolled into the study.

**Results:**

The mean age of presentation was 54.4 ± 7.7 years and percentage of males were 62 (51.66%). In this cohort, 72(60%) were treated with empagliflozin, 34(28.33%) with dapagliflozin and 14(11.66%) with canagliflozin. Twenty patients had genital mycotic infection and 4 had urinary tract infection. Female patients had higher incidence of infections than male patients with no statistically significant difference (P = ns). We did not find any significant correlation between age of the patient, gender, duration of disease and treatment, HbA1c, different types and dose of SGLT2i used with the incidence of genital mycotic infections (P = ns).

**Conclusion:**

We need to counsel the patients before starting SGLT2i regarding possible chance of getting genitourinary infection, proper genital hygiene, drinking plenty of water and consulting the doctor if any symptoms at the earliest.

## Introduction

Diabetes mellitus (DM) is a chronic metabolic disorder, due to either an absolute or relative insufficiency of insulin, its action or both. This leads to uncontrolled hyperglycaemia. Infection is one of the important health challenges in patients with DM increasing morbidity and mortality. Diabetic patients are more prone to develop infections compared to non-diabetic, usually twice likely as non-diabetics 1. We know that there is greater risk of genitourinary infections in DM patients. Microbial agents responsible are fungi, viruses, bacteria, and parasites. There is usually overlap of genital and urinary infections in DM patients. Males usually develop balanoposthitis and females develop vulvovaginal infections^2^. According to the literature Indian diabetic women are most likely to develop vulvovaginal candidiasis (VVC) 3. In diabetic men Candida balanitis is the most common infection reported especially if they are uncircumcised 4. According to a US study on management of risk factors for infection, women and men with T2DM were 2.3- and 1.9-times more prone to develop genitourinary infection than women and men without diabetes 5. *C. albicans* is the frequent causative organism causing balanitis in men, and *C. glabrata* is the main pathogen in women with genital mycotic infections 6,7. Because of high sugar concentration in urine in uncontrolled DM patients, the urinary tract becomes a favoured area for multiplication of bacteria and fungi. The main symptoms of infections are itching, burning micturition, yellow white discharge, and dyspareunia. Management involves strict control of blood glucose and local application of antifungal ointments and/or oral antifungal and antibiotics 8.

Sodium glucose co transporter 2 inhibitors (SGLT2i) are a new group of oral hypoglycaemic drugs approved for treatment of type 2 DM. SGLT2i acts mainly on proximal tubules of kidneys where they inhibit glucose re absorption, thus increasing urinary glucose excretion^9^,^10^.These drugs are relatively safe and well tolerated but since they induce glycosuria, it may results in growth of microbials in the genital tract thus favouring mycotic infections and urinary tract infections (UTIs) 11,12. Previous studies have shown that patients on SGLT2i have 2 to 3-fold higher risk of genital mycotic infections (∼8–10%) compared to patients receiving placebo. (3–5%) 13,14,15. In most of these patients' infections are mild and resolve spontaneously or some patients may require local antifungal treatment. The SGLT2i available in India are empagliflozin, dapagliflozin and canagliflozin. Not many studies have been done about the prevalence of genital and urinary tract infections due to SGLT2i, more so in south India. Thus, we did a retrospective analysis to find out the prevalence of genitourinary infections in diabetes mellitus patients receiving SGLT2 inhibitors.

## Methods

This was retrospective study in the department of endocrinology, tertiary care hospital in south India. We pooled the data of all patients with T2D receiving SGLT2i. All patients who were taking SGLT2i for at least 12-months, were included in the analysis. We also collected the demographic data such as age, sex, duration of diabetes, HbA1c etc. SGLT2i were usually added when patients failed to respond to metformin and glimepiride combination therapy and if their HbA1c level was more than 7. All the symptoms of genitourinary infections like itching, redness, white discharge, fever, burning micturition were recorded. Any past history of genitourinary infection was also recorded.

## Statistical analysis

We analysed the data using SPSS version 17.0 software. All the data regarding continuous variables were presented as means and ± SD. The relationship between two variables were determined by Pearson's Chi square test. P < 0.05 was considered statistically significant. We obtained institutional ethical board permission for retrospective analysis of data.

## Results

The patients' demographic features are shown in Table 1. Most were above 50 years of age with mean age of 54.4 ± 7.7 years. Of these 120 patients, 62 were males. Most of the patients had diabetes of more than 5 years with mean duration of 10.5±6.4 years. They had poor glycerine control with mean HbA1c 8.8±1.8%; and most HbA1c above 7. Empagliflozin was the most commonly used SGLT2i with 60% patients on it. Meanwhile 28.33% of the patients were on dapagliflozin and 11.66% patients on canagliflozin.

**Table 1:** showing demographic data of patients

Baseline characteristics	Number of patients (n =120)
Age in years	54.4 ± 7.7
Sex	
Male	62(51.66)
Female	58(48.33)
Mean duration of diabetes in years	10.5±6.4
Mean HbA1c	8.8±1.8
Class of SGLT2i	
Empagliflozin 10mg	40
Empagliflozin 25mg	32
Dapagliflozin 5mg	20
Dapagliflozin 10mg	14
Canagliflozin 100mg	14

Of 120 patients ,20(16.66%) had one or more episode of genital mycotic infection. Eight patients had recurrent genital mycotic infection. There was no gender difference in occurrence of genital mycotic infections. We had 11 female and 9 male patients who had genital infection. [table 2] Most patients' genital mycotic infection occurred within one year of starting treatment (84%). Empagliflozin was used in 60% of the patients with genital mycotic infection while dapagliflozin in 30%, and canagliflozin in 10 (P = ns). We did not find any correlation between age of the patient, gender, duration of disease and therapy, HbA1c, different types of SGLT2i with the prevalence of genital urinary infections [Table 3]. Most men had infection with *Candida albicans* while in women *Candida glabrata* was main pathogen. Urinary tract infection was seen in 4 patients, (3 female and one male). Escherichia coli was the isolated organism in all four patients. All the patients responded well to topical or oral antifungal and antibacterial treatment. Four patients (20%) gave a history of previous genital infection compared to 7 patients (7%) without genital infection.

**Table 2:** showing genital mycotic and urinary tract infection in study population

Type of infection	No of patients (120)
Mycotic infection (total)	20(16.6%)
Male	9(45%)
Female	11(55%)
Empagliflozin	12(60%)
Dapagliflozin	6(30%)
Canagliflozin	2(10%)
Urinary tract infection	4
Recurrent mycotic infection	8(6.66%)

**Table 3:** Comparison of different **patient's** characteristics with genital infection

Parameters	No of patient who got genital infection (n=20)	No infection (n=100)	P value (>0.05 =NS)
Age group			
35–50	9(45%)	46(46%)	NS
50–65	11(55%)	54(54%)	NS
Sex			
Male	9(45%)	53(53%)	NS
Female	11(55%)	47(47%)	NS
Duration of diabetes			
Less than 5 years	8(40%)	48(48%)	NS
More than 5 years	12(60%)	52(52%)	NS
HbA1c			
Less than 7	2(10%)	9(9%)	NS
More than 7	18(90%)	91(91%)	NS
SGLT2i			
Empagliflozin	12(16.6%)	60(83.33%)	NS
Dapagliflozin	6(17.6%)	28(82.3%)	NS
Canagliflozin	2(14.2%)	12(85.8)	NS
Duration of treatment			
Less than 1 year	16(84%)	85(85%)	NS
More than 1 year	4(16%)	15(15%)	NS

## Discussion

In our study 20(16.66%) patients developed genital mycotic infection while 4(3.33%) patients developed urinary tract infection. Other studies also reported similar incidence of genital mycotic infections.

A pooled analysis of different SGLT2i is shown in table 4. Empagliflozin studies by Zinman et al showed frequency of genital mycotic infection was 6.5% and 6.3% with 10mg and 25mg respectively. As per study by Kim et al incidence of genital infection was 4.2% and 3.6% with 10mg and 25mg respectively. In another study from India by Agarwal et al showed higher incidence of genital mycotic infection (27%). In our study frequency was 16.6%. Study by Prasanna Kumar et al involving 9439 patients showed prevalence of genital mycotic infection was 3.4% and 4.5% with canagliflozin 100mg and 300mg respectively. Meanwhile study by Bode et al using canagliflozin 100 and 300mg reported 14.5% prevalence of genital mycotic infection. As per our study prevalence was 14.2% which was similar to study by Bode et al.

**Table 4:** Showing various studies with SGLT2 inhibitor therapy and genitourinary infection

Type of SGLT2i	Study (author)	Study population	Dose of SGLT2i	Incidence of infection
Dapagliflozin	Yabe et al [15].	16664	10 mg	**2.46% - 4.99%**
	Johnsson et al [16].	4545	2.5 mg, 5mg 10mg	4.1% with 2.5 mg, 5.7%with 5 mg and, 4.8% with 10mg dose
Canagliflozin	Prasanna Kumar et al [17]	9439	100 mg or 300 mg	3.4% with 100mg dose and 4.5% with 300 mg dose
	Bode et al [18].	714	100 mg or 300 mg	14.5% with 100 mg and 14.45%with 300mg dose
Empagliflozin	Zinman et al [19].	6563	10 mg or 25 mg	6.5% with 10 mg dose and 6.3% with 25mg
	Kim et al [20].	2477	10 mg or 25 mg	4.2%with 10 mg dose and 3.6% with 25 mg
SGLT2i Empagliflozin Dapagliflozin- Canagliflozin-	Agarwal et al [21]	205(total) 104 39 62	10mg(n=74), 25mg(n=30) 5mg(n=8), 10mg(n=31) 100mg(n=60), 300mg(n=2)	27% 28.2% 20.9%
Empagliflozin- Dapagliflozin- Canagliflozin-	Present study	120(total) 72 34 14	10mg(n=40), 25mg(n=32) 5mg(n=20), 10mg(n=14) 100mg(n=14),	16.6% !7.6% 14.2%

A met analysis done on dapagliflozin reported that 4.1 to 5.7% patients treated with dapagliflozin developed genitourinary infection compared to 0.9% in placebo group. A study by Yabe et al involving 16664 patients reported 2,446 to 4.99% prevalence of genital mycotic infection. Whereas study by Johnson et al reported 4.1%, 5.7% and 4.8% prevalence of genital mycotic infection with 2.5mg,5mg and 10mg respectively. In most of studies involving dapagliflozin prevalence ranges from 2.5% to 13% with varied doses.

Poor genital hygiene may be reason for relatively higher percentage of patients developing genital infection in our cohort. A study by Goswami et al 22 showed that Indian women with T2 diabetes are more prone to develop vulvovaginal candidiasis (46%) compared to general population (23%). Though, there are few studies which reported lower incidence genital infections in Indians in hot and humid conditions, Indian patients with T2D may have enhanced risk of genital mycotic infections [20]. Therefore, we should be careful about the side effects of SGLT2i and monitor for them. Relatively higher proportion in males may be due to lack of circumcision. Various studies have shown that balanoposthitis is rare in circumcised men. Since most of south Indian population consume high carbohydrate containing diet, the resulting glucosuria may increase the chance of genital infection. Knowledge about the drug and its action found to be useful in reducing the incidence of genital infection. We should educate the patients about possibility of genital infection and how to prevent it. The measures to prevent infection are proper hydration, maintaining genital hygiene by washing the genital after urination, withholding SGLT2i if develop symptoms for short period and use of local anti fungals. We did not find any difference in frequency of genital mycotic infections with individual SGLT2i. Geerlings et al 12 reported that genitourinary infection is a class effect rather than individual drug effect. We don't have studies comparing the drugs in terms of genitourinary infections.

## Limitations

One main limitation of our study is small sample size. Since majority of our patients had poor glycaemic control, this may be confounding factor in increasing the incidence of genital infection thus falsely increasing the event rate. In our study there was no correlation between HbA1c and frequency of genital infection.

## Conclusion

The risk of urogenital infections is more among Indian patients with T2D on SGLT2i therapy. We need to counsel the patient before starting SGLT2i regarding possible chance of getting genitourinary infection, proper genital hygiene, drinking plenty of water and consult the doctor if develops any symptoms at the earliest.
